# Psychometric Properties of the Greek Version of the Claustrophobia Questionnaire

**DOI:** 10.3390/bs15081059

**Published:** 2025-08-05

**Authors:** Varvara Pantoleon, Petros Galanis, Athanasios Tsochatzis, Foteini Christidi, Efstratios Karavasilis, Nikolaos Kelekis, Georgios Velonakis

**Affiliations:** 1 Research Unit of Radiology and Medical Imaging, 2nd Department of Radiology, Attikon General University Hospital, School of Medicine, National and Kapodistrian University of Athens, 115 27 Athens, Greece; skaravasilis82@gmail.com (E.K.); kelnik@med.uoa.gr (N.K.); giorvelonakis@gmail.com (G.V.); 2 Clinical Epidemiology Laboratory, Faculty of Nursing, National and Kapodistrian University of Athens, 115 27 Athens, Greece; pegalan@nurs.uoa.gr; 3 Department of CT-MRI, “Hygeia” Hospital, 151 23 Athens, Greece; thanasis.tsochatzis@hotmail.com; 4 2nd Department of Psychiatry, School of Medicine, National and Kapodistrian University of Athens, 115 27 Athens, Greece; christidi.f.a@gmail.com; 5 Computational Neuroimaging Group, Trinity College Dublin, D08 NHY1 Dublin, Ireland; 6 School of Medicine, Democritus University of Alexandroupolis, 681 00 Alexandroupolis, Greece

**Keywords:** claustrophobia, Claustrophobia Questionnaire (CLQ), psychometric validation, Greek version, anxiety disorders, MRI anxiety, cross-cultural adaptation, reliability, validity

## Abstract

**Background**: Claustrophobia is defined as the fear of enclosed spaces, and it is a rather common specific phobia. Although the Claustrophobia Questionnaire (CLQ) is a valid questionnaire to measure claustrophobia, there have been no studies validating this tool in Greek. Thus, our aim was to translate and validate the CLQ in Greek. **Methods**: We applied the forward–backward translation method to translate the English CLQ into Greek. We conducted confirmatory factor analysis (CFA) to examine the two-factor model of the CLQ. We examined the convergent and divergent validity of the Greek CLQ by using the Fear Survey Schedule-III (FSS-III-CL), the NEO Five-Factor Inventory (NEO-FFI-NL-N), and the Spielberger’s State-Trait Anxiety Inventory (STAI). We examined the convergent validity of the Greek CLQ by calculating Pearson’s correlation coefficient between the CLQ scores and scores on FSS-III-CL, NEO-FFI-NL-N, STAI-S (state anxiety), and STAI-T (trait anxiety). We examined the divergent validity of the Greek CLQ using the Fisher r-to-z transformation. To further evaluate the discriminant validity of the CLQ, we calculated the average variance extracted (AVE) score and the Composite Reliability (CR) score. We calculated the intraclass correlation coefficient (ICC) and Cronbach’s alpha to assess the reliability of the Greek CLQ. **Results**: Our CFA confirmed the two-factor model of the CLQ since all the model fit indices were very good. Standardized regression weights between the 26 items of the CLQ and the two factors ranged from 0.559 to 0.854. The convergent validity of the Greek CLQ was very good since it correlated strongly with the FSS-III-CL and moderately with the NEO-FFI-NL-N and the STAI. Additionally, the Greek CLQ correlated more highly with the FSS-III-CL than with the NEO-FFI-NL-N and the STAI, indicating very good divergent validity. The AVE for the suffocation factor was 0.573, while for the restriction factor, it was 0.543, which are both higher than the acceptable value of 0.50. Moreover, the CR score for the suffocation factor was 0.949, while for the restriction factor, it was 0.954. The reliability of the Greek CLQ was excellent since the ICC in test–retest study was 0.986 and the Cronbach’s alpha was 0.956. **Conclusions**: The Greek version of the CLQ is a reliable and valid tool to measure levels of claustrophobia among individuals.

## 1. Introduction

Claustrophobia, defined as an irrational fear of confined spaces, can significantly impact sufferers’ daily lives. Individuals with claustrophobia often avoid situations such as using elevators or public transportation or even being in small rooms. These constraints can limit their activities and reduce their quality of life (Björkman-Burtscher, 2021; Vadakkan & Siddiqui, 2025). One of the most reliable and widely used tools for assessing claustrophobia is the Claustrophobia Questionnaire (CLQ). Originally developed by Radomsky et al. (2001), the CLQ has been validated in various cultural and linguistic populations, demonstrating its reliability and validity across different contexts (Radomsky et al., 2001, 2006; Van Diest et al., 2010). Numerous studies have demonstrated the robust psychometric properties of the CLQ. For example, the French and English versions of the CLQ have shown strong reliability and validity in both clinical and research settings (Radomsky et al., 2006). Similarly, the Dutch version of the CLQ has been validated and confirmed to possess good psychometric properties (Van Diest et al., 2010). Additionally, the CLQ has been successfully adapted for use in other languages and populations, such as the Spanish and the Swedish languages and populations, indicating its applicability across different cultural contexts (Palacios et al., 2003; Carlbring & Söderberg, 2001).

Claustrophobia is a common challenge for individuals undergoing imaging examinations, such as magnetic resonance imaging (MRI). In these latter scenarios, patients with claustrophobia may experience severe anxiety or panic due to the enclosed space, restricted movement, and loud noise of the MRI machine (Hudson et al., 2022a, 2022b). These factors intensify discomfort and can disrupt the imaging process (Hudson et al., 2022a). Research shows that about 10% of patients undergoing MRI report claustrophobic reactions, and 2–5% may even stop the procedure due to extreme anxiety (Carpenter et al., 2023). In practice, clinicians frequently resort to strategies such as pre-scan sedation, use of open MRI machines, or repeated scheduling, which may increase healthcare costs and delay diagnosis (Lawal et al., 2023).

Recent studies emphasize the need for early identification of claustrophobia before MRI scans. This allows for interventions such as medication or alternative imaging methods to be implemented (Rentmeester et al., 2022). Reliable assessment tools, like the Claustrophobia Questionnaire (CLQ), are vital for early recognition of claustrophobia and personalized management before and during the examination.

A recent study (Skalidis et al., 2024) highlights the necessity of adapting the CLQ for use in Greek-speaking populations, confirming the tool’s validity and reliability. This research underscores the need for a reliable instrument to assess claustrophobia in Greek patients, particularly before procedures that could trigger anxiety, ensuring appropriate preparation and support.

Reliable and valid assessment of claustrophobia is crucial for accurate diagnosis and effective treatment of the disorder. Despite the extensive international use of CLQ, there is a lack of data on the application and psychometric evaluation of the CLQ within the Greek population. Validating a questionnaire in a specific cultural context is crucial, as cultural and linguistic differences can influence the manifestation and reporting of symptoms. For instance, perceptions and attitudes towards confined spaces may vary significantly across cultures, affecting the validity of the questionnaire’s results. Additionally, validating the CLQ in Greek will contribute to cross-cultural psychology, providing data that will facilitate comparisons between different cultural populations (Mohammadi et al., 2019). Standardizing the CLQ in a Greek sample will improve the understanding and management of claustrophobia in Greece, providing a culturally sensitive tool that aligns with international research standards. This research will also offer valuable insights into the nature and extent of claustrophobia in the Greek population, enhancing therapeutic interventions and health policies (Karaivazoglou et al., 2021). Furthermore, it will aid in developing more comprehensive and inclusive psychological assessments that consider cultural nuances and variations in symptomatology (Kim & Kwon, 2020).

Therefore, considering that the validation of the CLQ in the local, Greek-speaking population is essential for the reliable and valid assessment of claustrophobia, this study aims to validate the CLQ in the Greek-speaking population, providing a valuable tool for clinicians and researchers.

## 2. Materials and Methods

### 2.1. Participants

We included 314 Greek-speaking individuals of both genders, aged between 18 and 70 years old. The inclusion criteria were Greek as the primary language; at least 3 years of education; and age >17. The exclusion criteria were major psychiatric disorders (e.g., major depression, schizophrenia, dementia, or substance use disorders). Additionally, we conducted a pilot study (*n* = 50) to examine the test–retest reliability of the Greek CLQ. We created an online version of the study questionnaire through Google Forms, and we posted it on Facebook to inform users of the purpose and the design of the study. Thus, we obtained a convenience sample. All questions on the online study questionnaire were mandatory, but individuals had the opportunity to terminate their participation at any time. Since all the questions were mandatory, we did not have missing data. There were no incentives for participation in our study. Confirmatory factor analysis requires at least 200 participants (Klein, 2016), and, thus, our sample covered this requirement.

Our study adhered to the principles outlined in the Declaration of Helsinki (General Assembly of the World Medical Association, 2014). The Ethics Committee of the Faculty of Medicine at the National and Kapodistrian University of Athens granted approval for our study protocol (724/25-10-2023). All participants gave their informed consent to participate in the study.

### 2.2. Procedure

We applied the forward–backward translation method to translate the English CLQ into Greek. Additionally, we followed the suggested procedure for cross-cultural adaptation of questionnaires (Sousa & Rojjanasrirat, 2011). In short, two independent scholars translated the English CLQ into Greek and reached a consensus. Then, two other scholars translated the Greek CLQ back into English and reached a consensus. We compared the final English CLQ with the original English CLQ and confirmed its linguistic accuracy. Afterward, we examined the face validity of the Greek CLQ by performing cognitive interviews (Meadows, 2021) with ten individuals. We did not identify linguistic issues, and no changes were necessary during this step. Participants confirmed the clarity of the 26 items. We obtained permission from CLQ developers to translate the tool into Greek and validate it in this language. Data collection occurred in January 2024.

### 2.3. Measures

**The Claustrophobia Questionnaire [CLQ** (Radomsky et al., 2001)**]:** The CLQ contains 26 items measuring levels of claustrophobia on a five-point Likert scale ranging from 0 (not at all anxious) to 4 (extremely anxious) (Radomsky et al., 2001). The CLQ comprises two factors: suffocation (14 items) and restriction (12 items). Total scores range from 0 to 104, with higher scores indicating higher levels of claustrophobia.

**The Fear Survey Schedule-III [FSS-III** (Wolpe & Lang, 1964)**]:** The FSS-III measures specific fears, including three items that assess claustrophobic fear (FSS-III-CL): “I am afraid of crowds,” “I am afraid of being in an elevator,” and “I am afraid of enclosed spaces.” Responses are on a five-point Likert scale ranging from 0 (not at all afraid) to 4 (extremely afraid). The FSS-III-CL score ranges from 0 to 12, with higher scores indicating higher levels of claustrophobic fear. We used the validated Greek version of the FSS-III-CL (Mellon, 2000), which showed a Cronbach’s alpha of 0.803 in our study.

**The NEO Five-Factor Inventory [NEO-FFI-NL** (Costa & McCrae, 1992)**]:** This inventory assesses five personality traits, but we used the 12 items measuring neuroticism. Responses are on a five-point Likert scale from 1 (strongly disagree) to 5 (strongly agree), with higher scores indicating higher levels of neuroticism. We used the validated Greek NEO-FFI-NL-N (Panayiotou et al., 2004), which had a Cronbach’s alpha of 0.824 in our study.

**The Spielberger’s State-Trait Anxiety Inventory [STAI** (Spielberger, 1983)**]:** The STAI includes 40 items measuring state (STAI-S) and trait (STAI-T) anxiety on a four-point Likert scale from 1 (never) to 4 (almost always). Scores range from 20 to 80, with higher scores indicating higher anxiety levels. We used the validated Greek STAI, which showed a Cronbach’s alpha of 0.934 for trait anxiety and 0.854 for state anxiety in our study (Fountoulakis et al., 2006).

### 2.4. Statistical Analysis

Categorical variables are presented as numbers (percentages), and continuous variables as means (standard deviations, SD). The Kolmogorov–Smirnov test was used to examine the distribution of continuous variables, and parametric statistics were applied following normal data distribution.

We conducted confirmatory factor analysis (CFA) to examine the previously reported factor structure of the CLQ in Greek. We employed CFA to examine the construct validity of the CLQ. Since CLQ data are on an ordinal scale, we used the weighted least squares method (Li, 2016). We chose the weighted least squares method over maximum likelihood estimation since the assumption of multivariate normality was violated due to our ordinal data. Since the weighted least squares method is more robust against violations of normality, it provides more accurate parameter estimates and standard errors. Developers of the CFA suggest a two-factor model of the questionnaire: 14 items load onto a suffocation factor, and 12 items load onto a restriction factor (Radomsky et al., 2001). We calculated the chi-squared/degree of freedom (x^2^/df), root mean square error of approximation (RMSEA), goodness of fit index (GFI), adjusted goodness of fit index (AGFI), Tucker–Lewis index (TLI), incremental fit index (IFI), normed fit index (NFI), and comparative fit index (CFI). We adopted criteria for fit from previous studies: x^2^/df < 5, RMSEA < 0.10, and for all other indices > 0.90 (Li, 2016; Martinaki et al., 2023). Moreover, we calculated standardized regression weights between the 26 items of the CLQ and the two factors. CFA was conducted using the AMOS version 21 (Amos Development Corporation, 2018).

If more than 85% of participants achieved the lowest (1) or highest possible score (5) on the 26 items of the CLQ, respectively, we considered ceiling or floor effects (Yusoff et al., 2021).

To further evaluate the discriminant validity of the CLQ, we calculated the average variance extracted (AVE) score and the Composite Reliability (CR) score. Acceptable values for the AVE score are higher than 0.50 (Fornell & Larcker, 1981), while, for the CR score, they are higher than 0.70 (Anderson & Gerbing, 1988; Fornell & Larcker, 1981).

We examined the convergent validity of the Greek CLQ by calculating Pearson’s correlation coefficient between the CLQ scores and scores on FSS-III-CL, NEO-FFI-NL-N, STAI-S (state anxiety), and STAI-T (trait anxiety). We examined the divergent validity of the Greek CLQ using the Fisher r-to-z transformation. In particular, we compared the correlations of the Greek CLQ with other measures of claustrophobia (FSS-III-CL), questionnaires assessing neuroticism (NEO-FFI-NL-N), state anxiety (STAI-S), and trait anxiety (STAI-T). Since the FSS-III-CL measures claustrophobic fear and the CLQ measures levels of claustrophobia, we expected a higher correlation between CLQ and FSS-III-CL than the correlation between CLQ and STAI.

To assess the reliability of the CLQ in the test–retest study, we employed the intraclass correlation coefficient (ICC). We utilized a two-way mixed-effects model with absolute agreement, calculating ICCs, 95% confidence intervals (CIs), and *p*-values. Excellent reliability is indicated by ICC values exceeding 0.90 (Koo & Li, 2016).

To evaluate the internal reliability of the Greek CLQ, we computed Cronbach’s alpha for all 26 items, as well as Cronbach’s alpha with individual items removed. A Cronbach’s alpha greater than 0.70 was deemed acceptable (Bland & Altman, 1997). Additionally, we determined corrected item–total correlations to gauge the Greek CLQ’s reliability, with values ≥0.30 considered acceptable (De Vaus, 2004).

The statistical threshold was set at *p*-value < 0.05. All previously reported analyses were conducted using IBM SPSS 21.0 (IBM Corp. Released 2012. IBM SPSS Statistics for Windows, Version 21.0. Armonk, NY, USA: IBM Corp.).

## 3. Results

### 3.1. Study Participants

Participants were 314 Greek-speaking individuals aged 18–70 years. The mean age was 39.3 years (SD 12.1), while the median age was 38 years. In our sample, 69.1% (*n* = 217) were female and 30.9% (*n* = 97) were male. Demographic characteristics of our sample are shown in Table 1.

Levels of claustrophobia were higher among females than males. In particular, the mean total CLQ score was 38.4 (SD; 23.2) and 24.4 (SD; 18.3) for females and males (*p*-value < 0.001), respectively. Moreover, the mean score for the suffocation factor was 14.8 (SD; 12.1) for females and 8.6 (SD; 9.5) for males (*p*-value < 0.001). The mean score for the restriction factor was 23.5 (SD; 13.2) for females and 15.6 (SD; 10.9) for males (*p*-value < 0.001). There were no significant correlations between age and CLQ score (r = 0.041, *p*-value = 0.468), suffocation factor score (r = 0.039, *p*-value = 0.494), or restriction factor score (r = 0.037, *p*-value = 0.516).

### 3.2. Factor Structure

**Figure 1:**
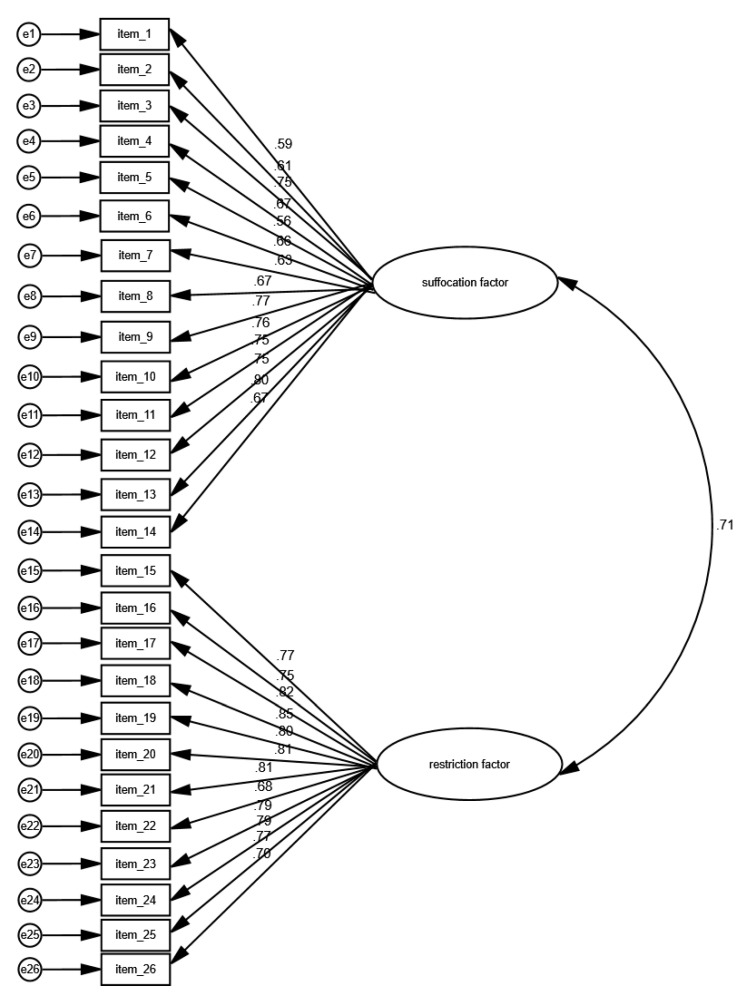
Confirmatory factor analysis of the Greek version of the Claustrophobia Questionnaire.

Figure 1 shows the results from the CFA for the Greek CLQ. All indices showed a good fit of the two-factor model. In particular, x^2^/df was 1.199, RMSEA was 0.025, GFI was 0.936, AGFI was 0.904, TLI was 0.989, IFI was 0.992, NFI was 0.955, and CFI was 0.992. Moreover, the correlation between the suffocation factor and the restriction factor was 0.71 (*p* < 0.001). Since the correlation between the two factors was high, there was probably an overlap between the items for the factors. The suffocation factor includes 14 items, while the restriction factor includes 12 items, and high correlations between some items are probable. Standardized regression weights between the 26 items of the CLQ and the two factors ranged from 0.559 to 0.854. Thus, the Greek CLQ confirmed the two-factor model structure of the original version of the questionnaire.

We did not find ceiling or floor effects on our data. To further evaluate the discriminant validity of the CLQ, we calculated the AVE score and the CR score for the two factors. The AVE for the suffocation factor was 0.573, while, for the restriction factor, it was 0.543, which are both higher than the acceptable value of 0.50. Moreover, the CR score for the suffocation factor was 0.949, while, for the restriction factor, it was 0.954, which are higher than the acceptable value of 0.70. Additionally, the AVE for each construct is greater than the squared correlations with other constructs. Thus, the discriminant validity of the Greek version of the CLQ was acceptable.

### 3.3. Convergent Validity

Table 2 illustrates the correlations between the Greek CLQ and other questionnaires used in the study. Pearson’s correlation coefficient being between 0 and 0.3 denotes a small correlation, between 0.31 and 0.70 denotes a moderate correlation, and between 0.71 and 1.0 denotes a strong correlation. Therefore, a strong correlation was observed between the Greek CLQ and the FSS-III-CL (r = 0.744, *p* < 0.001), while a moderate correlation was found with the NEO-FFI-NL-N (r = 0.394, *p* < 0.001), the STAI-S (r = 0.302, *p* < 0.001), and the STAI-T (r = 0.383, *p* < 0.001). A similar pattern of correlations was identified when CLQ suffocation and restriction factors were considered separately. Thus, we found a strong correlation between the suffocation factor and the FSS-III-CL (r = 0.740, *p* < 0.001), and a moderate correlation between the suffocation factor and the NEO-FFI-NL-N (r = 0.367, *p* < 0.001), the STAI-S (r = 0.346, *p* < 0.001), and the STAI-T (r = 0.371, *p* < 0.001). Additionally, our findings showed a moderate correlation between the restriction factor and the FSS-III-CL (r = 0.630, *p* < 0.001), the NEO-FFI-NL-N (r = 0.356, *p* < 0.001), and the STAI-T (r = 0.333, *p* < 0.001). Also, we found a small correlation between the restriction factor and the STAI-S (r = 0.214, *p* < 0.001).

### 3.4. Divergent Validity

The Greek CLQ correlated more highly with the FSS-III-CL than with the NEO-FFI-NL-N [z = 6.77, *p* < 0.001], the STAI-S [z = 8.08, *p* < 0.001], and the STAI-T [z = 6.93, *p* < 0.001]. The suffocation factor of the CLQ correlated more highly with the FSS-III-CL than with the NEO-FFI-NL-N [z = 7.05, *p* < 0.001], the STAI-S [z = 7.35, *p* < 0.001], and the STAI-T [z = 6.99, *p* < 0.001]. The restriction factor of the CLQ correlated more highly with the FSS-III-CL than with the NEO-FFI-NL-N [z = 4.60, *p* < 0.001], the STAI-S [z = 6.53, *p* < 0.001], and the STAI-T [z = 4.93, *p* < 0.001].

### 3.5. Reliability Analysis

The ICC for the CLQ score in the test–retest study was 0.986 (95% CI = 0.975 to 0.992, *p* < 0.001). Additionally, the ICC for the suffocation factor was 0.964 (95% CI = 0.936 to 0.979, *p* < 0.001), and the restriction factor was 0.986 (95% CI = 0.975 to 0.992, *p* < 0.001). Therefore, the reliability of the CLQ was excellent.

The Cronbach’s alpha for the CLQ score, the suffocation factor, and the restriction factor was 0.956, 0.926, and 0.949, respectively. The Cronbach’s alpha for the CLQ score did not increase after the elimination of each single item. Corrected item–total correlations ranged from 0.494 to 0.770 (Table 3). Thus, the internal consistency reliability of the CLQ was excellent.

## 4. Discussion

The goal of this study was to validate the Greek version of the CLQ by assessing its psychometric properties in a Greek sample. The findings suggest that the Greek version of the CLQ demonstrates robust psychometric properties, aligning with prior validations conducted in different languages and cultural settings (Fariborz, 2021; Panayiotou et al., 2004; Radomsky et al., 2001; Carlbring & Söderberg, 2001; Van Diest et al., 2010).

A claustrophobia questionnaire could be an effective tool in clinical practice, helping to assess the severity of the condition and identify specific triggers. By using such questionnaires, clinicians can develop personalized treatment plans that address the patient’s unique needs (Björkman-Burtscher, 2021). Cognitive–behavioral therapy (CBT) or gradual exposure therapy are commonly used approaches that aim to reduce anxiety by slowly desensitizing the individual to their fears, helping them regain control over their life (Kampmann et al., 2016; Radomsky et al., 2001).

Construct validity of the Greek CLQ was evaluated using CFA. An analysis of the questionnaire confirmed its two-factor structure (suffocation and restriction). The fit indices (x^2^/df < 5, RMSEA < 0.10, GFI, AGFI, TLI, IFI, NFI, and CFI > 0.90) were within acceptable ranges, indicating that the Greek version maintains the structural integrity of the original CLQ (Radomsky et al., 2001). The significant correlation between the two factors further supports the original bidimensional framework. Convergent validity was established by examining the Pearson correlation coefficients between the CLQ and other established measures of anxiety and personality traits, including the Fear Survey Schedule-III (FSS-III-CL), the NEO Five-Factor Inventory (NEO-FFI-NL), and the Spielberger State-Trait Anxiety Inventory (STAI). The CLQ showed strong correlations with the FSS-III-CL and moderate correlations with the NEO-FFI-NL and STAI, confirming its excellent convergent validity (Costa & McCrae, 1992; Mellon, 2000; Radomsky et al., 2006). Divergent validity was assessed using Fisher’s r-to-z transformation, comparing the correlation strengths between the CLQ and FSS-III-CL with those between the CLQ and NEO-FFI-NL and STAI. The stronger correlations with the FSS-III-CL compared to the other measures confirmed the CLQ’s specificity in measuring claustrophobia distinctly from general anxiety and personality traits (Panayiotou et al., 2004; Wolpe & Lang, 1964). The reliability of the Greek CLQ was assessed through internal consistency and test–retest reliability measures. The overall Cronbach’s alpha was 0.956, with the suffocation factor at 0.926 and the restriction factor at 0.949, indicating excellent internal consistency. The intraclass correlation coefficient for the overall scale was 0.986, demonstrating high test–retest reliability and stability over time (Koo & Li, 2016). These results align with those from previous CLQ validations in different languages, further supporting its reliability across diverse cultural contexts (Costa & McCrae, 1992; Panayiotou et al., 2004; Carlbring & Söderberg, 2001; Spielberger, 1983; Van Diest et al., 2010).

A claustrophobia assessment tool can be extremely useful for patients scheduled for an MRI scan, as it helps identify those who are more likely to experience high anxiety during the procedure. When these patients are identified ahead of time, healthcare professionals can take steps like administering sedative medications or opting for an open MRI scanner to make the experience more manageable (Iwan et al., 2021; Snoek et al., 2021; Zhou et al., 2023). This not only makes the process easier for the patient, it also helps avoid cancelations or delays, ultimately leading to better overall patient care (Al-Shemmari et al., 2022; Iwan et al., 2021). There is a gap in Greek clinical practice regarding the systematic assessment of claustrophobia, particularly in settings such as MRI procedures, surgical preparation, or emergency care (Napp et al., 2021). In the absence of a validated Greek-language instrument, clinicians often rely on informal observation or self-report, which may overlook individuals with moderate but clinically relevant symptoms. The Greek version of the CLQ offers a standardized and psychometrically robust tool to support early detection, treatment planning, and improved patient outcomes.

Beyond its clinical value, such a tool holds significant importance in research settings. Being able to measure claustrophobia levels allows researchers to select appropriate participants for functional magnetic resonance imaging (fMRI) studies focusing on claustrophobia, gaining insights into brain regions such as the amygdala and insular cortex, which are involved in fear and spatial awareness (Mutlu & Arik, 2023; Pfurtscheller et al., 2021). Understanding these neural mechanisms is crucial for developing targeted interventions.

Furthermore, an assessment tool allows researchers to measure the effectiveness of different therapeutic interventions, such as CBT, relaxation techniques, or virtual reality exposure. Using fMRI to track changes in brain activity, researchers may evaluate how these interventions impact claustrophobic responses. Studies have found that patients who practice breathing control or focus on positive thoughts during MRI scans show reduced activity in brain areas linked to anxiety (Dostál et al., 2024; Hassan Kariri & Almubaddel, 2024). Thus, these tools provide a vital baseline for comparing brain activity before and after various coping strategies (Elias et al., 2022).

While this study achieves promising results, it has a few limitations that should be recognized. Firstly, as with all self-report instruments, there is a potential for response bias, as participants may overestimate or underestimate their symptoms due to social desirability, misunderstanding, or personal interpretation of the items. Secondly, cultural differences may influence the interpretation of claustrophobic experiences and responses, and while the CLQ has been validated in various contexts, subtle cultural nuances may still affect the outcomes. Moreover, we did not examine the criterion validity of the CLQ. Additionally, selection bias is probable since we used online recruitment. For instance, most of our participants were young females (69.1% with a mean age of 39.3 years). Further studies with random and more representative samples should be carried out. Last but not least, the cross-sectional design of the study precludes assessing changes in CLQ sensitivity over time, which longitudinal studies could do.

As a result of the validation of the Greek version of the CLQ for Greek-speaking populations, a reliable and valid tool for the assessment of claustrophobia is now available. This instrument can be used effectively in both clinical settings and research studies to diagnose and evaluate claustrophobia, thereby improving treatment outcomes and contributing to the field of cross-cultural psychology. The strong psychometric properties of the Greek CLQ support its use in comparative studies across different cultural groups, enhancing our understanding of how claustrophobia manifests and is experienced globally (Fariborz, 2021; Kampmann et al., 2016; Kim & Kwon, 2020).

While the psychometric structure of the CLQ has been confirmed in various populations, cultural factors may influence how claustrophobia is understood and expressed. In Greek culture, enclosed or crowded spaces—such as small elevators, public offices, or even religious settings—can elicit anxiety linked not only to physical discomfort but also to social pressure or lack of control. Additionally, mental health stigma in some Greek communities may lead individuals to underreport fear-related symptoms. Although the CLQ was well accepted by participants in this study, it is not yet widely recognized by Greek clinicians or the general public. Future research should explore awareness and acceptance of the instrument in clinical settings and assess whether further cultural adaptation or outreach is needed to enhance its practical use in Greece (Economou et al., 2020; Martinaki et al., 2023; Porfyri et al., 2022).

Future research should further examine the criterion validity of the Greek CLQ using structured clinical interviews and explore its sensitivity to change in longitudinal treatment settings.

## 5. Conclusions

A number of excellent psychometric properties have been demonstrated by the Greek version of the CLQ, including construct validity, convergent and divergent validity, and reliability. These findings are consistent with previous validations of the CLQ in other languages and cultural contexts. Consequently, the Greek CLQ is a valuable tool for clinicians and researchers, aiding in the accurate assessment and treatment of claustrophobia. Future research should explore the application of the CLQ in diverse populations to further validate its use and enhance its applicability across various cultural settings.

## Figures and Tables

**Table 1:** Demographic characteristics.

Characteristics	Ν	%
Gender		
Females (N/%)	217	69.1
Males (N/%)	97	30.9
Age (mean, standard deviation)	39.3	12.1
Age (median, interquartile range)	38.0	16.0

**Table 2:** Pearson’s correlation coefficients between the Greek CLQ and other questionnaires (N = 314).

	Claustrophobia Questionnaire (CLQ)	CLQ: Suffocation Factor	CLQ: Restriction Factor
Claustrophobia Subscale of the Fear Survey Schedule-III	0.744 ***	0.740 ***	0.630 ***
Neuroticism Subscale of the Dutch NEO Five-Factor Inventory	0.394 ***	0.367 ***	0.356 ***
Spielberger’s State-Trait Anxiety Inventory for State Anxiety	0.302 ***	0.346 ***	0.214 ***
Spielberger’s State-Trait Anxiety Inventory for Trait Anxiety	0.383 ***	0.371 ***	0.333 ***

*** *p* < 0.001.

**Table 3:** Reliability analysis for the Claustrophobia Questionnaire.

Items	Corrected Item–Total Correlation	Cronbach’s Alpha If Item Deleted
1	0.494	0.956
2	0.516	0.956
3	0.671	0.955
4	0.581	0.956
5	0.495	0.956
6	0.598	0.956
7	0.602	0.955
8	0.574	0.956
9	0.710	0.954
10	0.652	0.955
11	0.693	0.954
12	0.724	0.954
13	0.729	0.954
14	0.586	0.955
15	0.767	0.954
16	0.770	0.954
17	0.711	0.954
18	0.691	0.954
19	0.716	0.954
20	0.674	0.955
21	0.738	0.954
22	0.659	0.955
23	0.739	0.954
24	0.662	0.955
25	0.700	0.954
26	0.716	0.954

## Data Availability

Data will be made available on request. For requesting data, please write to the corresponding author.
